# Lymphocyte-Related Immunological Indicators for Stratifying *Mycobacterium tuberculosis* Infection

**DOI:** 10.3389/fimmu.2021.658843

**Published:** 2021-06-30

**Authors:** Ying Luo, Ying Xue, Guoxing Tang, Yimin Cai, Xu Yuan, Qun Lin, Huijuan Song, Wei Liu, Liyan Mao, Yu Zhou, Zhongju Chen, Yaowu Zhu, Weiyong Liu, Shiji Wu, Feng Wang, Ziyong Sun

**Affiliations:** ^1^ Department of Laboratory Medicine, Tongji Hospital, Tongji Medical College, Huazhong University of Science and Technology, Wuhan, China; ^2^ Department of Immunology, School of Basic Medicine, Tongji Medical College, Huazhong University of Science and Technology, Wuhan, China; ^3^ Department of Epidemiology and Biostatistics, Key Laboratory of Environmental Health of Ministry of Education, School of Public Health, Tongji Medical College, Huazhong University of Science and Technology, Wuhan, China; ^4^ Department of Laboratory Medicine, Zhejiang Provincial People’s Hospital, People’s Hospital of Hangzhou Medical College, Hangzhou, China

**Keywords:** lymphocyte, immunological biomarkers, immunodiagnostic model, active tuberculosis, latent tuberculosis infection, differential diagnosis

## Abstract

**Background:**

Easily accessible tools that reliably stratify *Mycobacterium tuberculosis* (MTB) infection are needed to facilitate the improvement of clinical management. The current study attempts to reveal lymphocyte-related immune characteristics of active tuberculosis (ATB) patients and establish immunodiagnostic model for discriminating ATB from latent tuberculosis infection (LTBI) and healthy controls (HC).

**Methods:**

A total of 171 subjects consisted of 54 ATB, 57 LTBI, and 60 HC were consecutively recruited at Tongji hospital from January 2019 to January 2021. All participants were tested for lymphocyte subsets, phenotype, and function. Other examination including T-SPOT and microbiological detection for MTB were performed simultaneously.

**Results:**

Compared with LTBI and HC, ATB patients exhibited significantly lower number and function of lymphocytes including CD4^+^ T cells, CD8^+^ T cells and NK cells, and significantly higher T cell activation represented by HLA-DR and proportion of immunosuppressive cells represented by Treg. An immunodiagnostic model based on the combination of NK cell number, HLA-DR^+^CD3^+^ T cells, Treg, CD4^+^ T cell function, and NK cell function was built using logistic regression. Based on receiver operating characteristic curve analysis, the area under the curve (AUC) of the diagnostic model was 0.920 (95% CI, 0.867-0.973) in distinguishing ATB from LTBI, while the cut-off value of 0.676 produced a sensitivity of 81.48% (95% CI, 69.16%-89.62%) and specificity of 91.23% (95% CI, 81.06%-96.20%). Meanwhile, AUC analysis between ATB and HC according to the diagnostic model was 0.911 (95% CI, 0.855-0.967), with a sensitivity of 81.48% (95% CI, 69.16%-89.62%) and a specificity of 90.00% (95% CI, 79.85%-95.34%).

**Conclusions:**

Our study demonstrated that the immunodiagnostic model established by the combination of lymphocyte-related indicators could facilitate the status differentiation of MTB infection.

## Introduction

Tuberculosis (TB) remains a major global health issue as a leading infectious disease caused by *Mycobacterium tuberculosis* (MTB) infection (1). It was reported that there were around 10 million cases and 1.5 million deaths in 2019 (2). Most subjects suffered with MTB infection stay clinically asymptomatic which is called latent TB infection (LTBI). A relatively small proportion of these individuals would develop to active TB (ATB) during their life (3, 4). TB control strategies largely focus on identification and treatment of people with ATB. Accurate and early diagnosis could minimize therapy period and maximize quality of life. Therefore, developing novel biomarkers for TB diagnostics with satisfactory value has become a priority for TB control.

To date, ATB diagnosis mainly relies on either insensitive (acid fast bacilli smears) or time consuming (mycobacterial culture) methods (5). The clinical use of these approaches often leads to defer initiation of therapy. Molecular methods such as GeneXpert MTB/RIF and GeneXpert MTB/RIF Ultra have begun to overcome some of these barriers (6–8). However, such tests cannot show sufficient advantages due to their suboptimal sensitivity that cannot meet clinical needs (9). Besides, they are unable to differentiate live from dead mycobacteria, and remain prohibitively expensive to operate. Interferon gamma release assays, including QuantiFERON-TB Gold In-Tube based on enzyme-linked immunosorbent assay and T-SPOT based on enzyme-linked immune-spot assay, were availably used to detect MTB infection (10–12). Nevertheless, both of these two methods could not distinguish between ATB and LTBI, while were also not recommended for ATB diagnosis especially in area with high TB burden (13).

Meanwhile, several studies described the utility of T cell receptor beta variable from peripheral blood for diagnosing MTB infection (14, 15). Howbeit, the current validation is limited and further exploration is needed. Multiple limitations registered by conventional tests of etiology hurdles to the timely diagnosis of disease and contribute to promote clinical progression as well as continued transmission. Recent advances in genomics (16, 17), transcriptomics (18–20), proteomics (21–23), and metabolomics (24–26) have effectively facilitated the diagnosis of TB. But these emerging methods often require prohibitively complex equipment and operations, which hinder their promotion of clinical applications. Meanwhile, most investigations in this area are preliminary. The results regarding clinical diagnostic value of these approaches were usually obtained in small sample populations or regions with limited incidence, and have not been verified by multiple centers and large sample sizes.

Besides, previous work has reported the low number of lymphocytes in TB patients (27). In addition, several studies have identified the specific characteristics of the immunophenotype in TB patients (28, 29). Furthermore, our team has previously introduced a novel method-lymphocyte function assay for evaluating lymphocyte function (30, 31). The test could reflect the activation, chemotaxis, and cytotoxicity of lymphocytes through the percentage of IFN-γ released under PMA/ionomycin stimulation (32). We have verified its diagnostic and prognostic value among a variety of disease models including lymphoma (33), kidney transplantation (31), and carbapenem-resistant organism infection (34). Up to now, there are few investigations of lymphocyte function assay in the area of TB diagnosis. Therefore, it is necessary to conduct a more comprehensive assessment of TB patients by combining the number, phenotype, and function of lymphocytes. The present study aims to clarify lymphocyte-related immune signatures of individuals under different status of MTB infection and investigate the diagnostic role of these indicators for the distinguishment between ATB, LTBI, and healthy controls (HC).

## Methods

### Study Design

The present study was performed at Tongji Hospital from January 2019 to January 2021. Adult participants with age equal or more than 18 years were consecutively enrolled to the study. ATB was diagnosed by the identification of MTB in sputum or bronchoalveolar lavage fluid based on mycobacterial culture or GeneXpert MTB/RIF with symptoms compatible of ATB including prolonged cough, chest pain, weakness or fatigue, weight loss, fever, and night sweats. LTBI was defined by positive T-SPOT result without symptomatic, microbiological, or radiological evidences of ATB as well as the history of TB (**Supplementary Figure 1**). Individuals with negative T-SPOT results and without any evidence of suspected ATB or other diseases were categorized as HC. Subjects with HIV infection or receiving anti-TB treatment for more than 2 weeks were excluded from the study. Besides, patients with other infectious diseases, tumors, and autoimmune diseases were excluded from this study. Lymphocyte-related immune profile including lymphocyte subsets, lymphocyte phenotype, and lymphocyte function was analyzed among ATB, LTBI, as well as HC. This study was approved by the ethics committee of Tongji Hospital, Tongji Medical College, Huazhong University of Science and Technology.

### Lymphocyte Subsets

**Figure 1:**
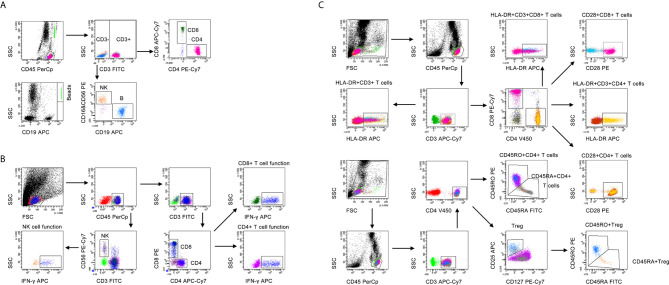
The gating strategies for **(A)** lymphocyte subset analysis, **(B)** lymphocyte function, and **(C)** lymphocyte phenotype analysis.

Heparinized peripheral blood was collected for performing lymphocyte subset analysis. The percentages and numbers of CD4^+^ T cells, CD8^+^ T cells, NK cells, and B cells were determined by using TruCOUNT tubes and BD Multitest 6-color TBNK Reagent Kit (BD Biosciences, San Jose, CA, USA) according to the manufacturer’s instructions. A volume of 50 µl peripheral blood was labeled with 6-color TBNK antibody cocktail for 20 min in room temperature. After adding 450 µl of FACS Lysing Solution, samples were analyzed with FACSCanto flow cytometer. Cells with positive CD45 expression and with low side scatter were gated as lymphocytes. TruCOUNT beads were gated based on side scatter and fluorescence intensity. CD3^+^ cells in lymphocyte gate were defined as total T cells. CD3^+^CD4^+^CD8^-^ and CD3^+^CD4^-^CD8^+^ cells were respectively defined as CD4^+^ T cells and CD8^+^ T cells. CD16^+^CD56^+^ cells and CD19^+^ cells in CD3^-^ cells were respectively defined as NK cells and B cells. The gating strategies for lymphocyte subsets analysis was shown in **Figure 1A**.

### Lymphocyte Function

Lymphocyte function assay was performed under PMA/ionomycin-stimulation as introduced previously (31). The operation was described as the following: (1) 100 µl of whole peripheral blood was diluted with 400 µl of IMDM medium (Gibco, Grand Island, NY, USA, cat 31980-030, plus 25mM HEPES and 3.024g/L Sodium Bicarbonate); (2) the diluted whole peripheral blood was incubated in the presence of Leukocyte Activation Cocktail (Becton Dickinson GolgiPlug™) for 4 h; (3) the cells were labeled with antibodies including anti-CD45, anti-CD3, anti-CD4, anti-CD8, and anti-CD56 for 20 minutes at room temperature; (4) the cell were fixed and permeabilized; (5) the cells were stained with intracellular anti-IFN-γ antibody; and (6) the cells were analyzed with FACSCanto flow cytometer. The percentages of IFN-γ^+^ cells in various cell subsets were defined as the function of them. Specially, the percentage of IFN-γ^+^ cells in CD3^+^CD4^+^CD8^-^ cells was regarded as CD4^+^ T cell function; the percentage of IFN-γ^+^ cells in CD3^+^CD4^-^CD8^+^ cells was regarded as CD8^+^ T cell function; the percentage of IFN-γ^+^ cells in CD3^-^CD56^+^ cells was regarded as NK cell function. The gating strategies for lymphocyte function assay was shown in **Figure 1B**.

### Lymphocyte Phenotype

Heparinized peripheral blood was collected for performing lymphocyte phenotype analysis. The following monoclonal antibodies were added to 100 µl of whole blood: anti-CD45, anti-CD3, anti-CD4, anti-CD8, anti-CD25, anti-CD127, anti-CD28, anti-HLA-DR, anti-CD45RA, and anti-CD45RO (BD Biosciences, San Jose, CA, USA). Isotype controls with irrelevant specificities were included as negative controls. Cell suspensions were incubated for 20 min at room temperature. The cells were washed and resuspended in 200 μl of phosphate buffer saline after lysing red blood cells. Then, the cells were analyzed with FACSCanto flow cytometer. The gating strategies for lymphocyte phenotype analysis was shown in **Figure 1C**.

### Statistical Analysis

Continuous variables were presented as mean ± standard deviation (SD) or median (interquartile range, IQR). The comparison between continuous variables was performed using T-test if the continuous value is normal distribution and homogeneity of variance or Mann-Whitney *U* test if not. Categorical variables were presented as numbers (percentages) and compared using Chi-square test or Fisher’s exact test. A two-tailed *p*-value less than 0.05 was considered statistically significant. For the establishment of immunodiagnostic model, indicators with statistical difference were selected and taken as candidates in multivariable logistic regression. Then, the regression equation (diagnostic model) was obtained. The regression coefficients of the model were regarded as the weights for the respective variables, and a score for each participant was calculated. Receiver operating characteristic (ROC) curve was plotted to evaluate the diagnostic performance of various indicators. Area under the curve (AUC), sensitivity, specificity, positive predictive value (PPV), negative predictive value (NPV), positive likelihood ratio (PLR), negative likelihood ratio (NLR), and accuracy as well as the corresponding 95% confidence interval (CI) were calculated. Z statistic was used for the comparison between AUCs with the procedure of Delong et al. (35). Data were analyzed using IBM SPSS 25.0 (SPSS Inc. Chicago, IL, USA), GraphPad Prism 8.0 (GraphPad Software, Inc. La Jolla, USA), MedCalc version 11.6 (MedCalc, Mariakerke, Belgium), and R 4.0.2 program (R Core Team).

## Results

### Participant Characteristics

A total of 171 subjects including 54 ATB, 57 LTBI, and 60 HC were consecutively enrolled from January 2019 to January 2021 at Tongji Hospital. The demographic and clinical manifestation of all participants were summarized in **Table 1**. There was no significant difference in scale of age and gender between these three groups. The median age was around 51 years. Males were predominant in all groups.

**Table 1:** Demographic and clinical characteristics of included subjects.

Variables	ATB (n = 54)	LTBI (n = 57)	HC (n = 60)
Age, years	51 (33-62)	51 (35-66)	52 (35-68)
Sex, male, %	31 (57.41%)	28 (49.12%)	34 (56.67%)
TB history	12 (22.22%)	0 (0%)	0 (0%)
Underlying condition or illness			
Diabetes mellitus	3 (5.56%)	3 (5.26%)	0 (0%)
End-stage renal disease	2 (3.7%)	2 (3.51%)	0 (0%)
Liver cirrhosis	2 (3.7%)	1 (1.75%)	0 (0%)
Positive mycobacterial culture	45 (83.33%)	N/A	N/A
Positive GeneXpert MTB/RIF	39 (72.22%)	N/A	N/A

ATB, active tuberculosis; LTBI, latent tuberculosis infection; HC, healthy controls; TB, tuberculosis; N/A, not applicable. Data were presented as medians (25th-75th percentiles) or numbers (percentages).

### Lymphocyte Subsets in ATB, LTBI, and HC

**Figure 2:**
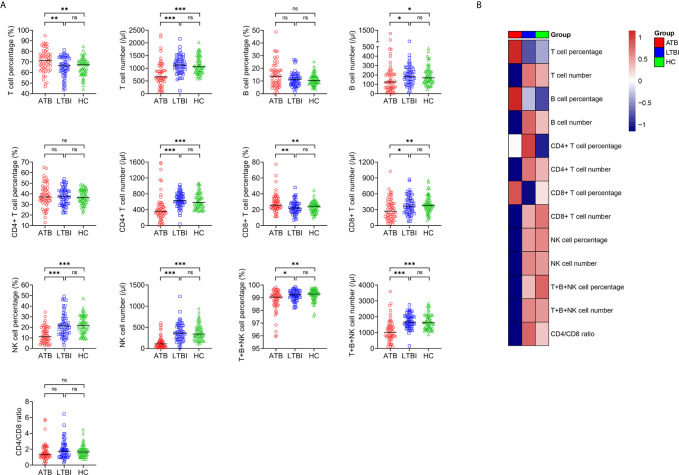
The results of lymphocyte subsets in ATB, LTBI, and HC. **(A)** Scatter plots showing the results of lymphocyte subsets in ATB (n = 54), LTBI (n = 57), and HC (n = 60). Horizontal lines indicate the median. *P < 0.05, **P < 0.01, ***P < 0.001, ns, no significance (Mann-Whitney *U* test). **(B)** Heatmap showing the results of lymphocyte subsets in ATB group, LTBI group, and HC group. Each rectangle indicates the median result of a group. ATB, active tuberculosis; LTBI, latent tuberculosis infection; HC, healthy controls.

We performed lymphocyte subset analysis among ATB patients, LTBI individuals, and HC. It was observed that compared with LTBI individuals, ATB patients showed significantly lower T cell number, B cell number, CD4^+^ T cell number, CD8^+^ T cell number, NK cell percentage, NK cell number, total percentage of T cells, B cells and NK cells (T+B+NK cell percentage), total number of T cells, B cells and NK cells (T+B+NK cell number), and higher T cell percentage, CD8^+^ T cell percentage (**Figure 2**). There was no significant difference in B cell percentage, CD4^+^ T cell percentage, and CD4/CD8 ratio between these two groups.

For the comparison between ATB group and HC group. T cell percentage and CD8^+^ T cell percentage were significantly higher, whereas T cell number, B cell number, CD4^+^ T cell number, CD8^+^ T cell number, NK cell percentage, NK cell number, T+B+NK cell percentage, and T+B+NK cell number were significantly lower in ATB patients than those in HC. No significant difference in B cell percentage, CD4^+^ T cell percentage, and CD4/CD8 ratio was found between ATB and HC (**Figure 2**). No significant differences in all indicators among lymphocyte subset analysis were observed in between LTBI and HC (**Figure 2**).

### Lymphocyte Phenotype in ATB, LTBI, and HC

**Figure 3:**
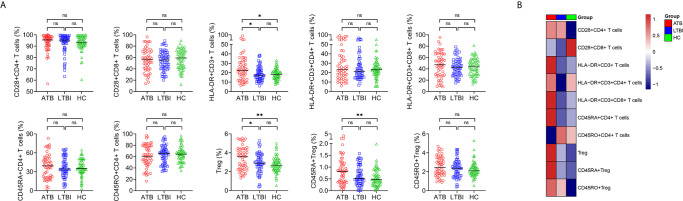
The results of lymphocyte phenotype in ATB, LTBI, and HC. **(A)** Scatter plots showing the results of lymphocyte phenotype in ATB (n = 54), LTBI (n= 57), and HC (n = 60). Horizontal lines indicate the median. *P < 0.05, **P < 0.01, ns, no significance (Mann-Whitney *U* test). **(B)** Heatmap showing the results of lymphocyte phenotype in ATB group, LTBI group, and HC group. Each rectangle indicates the median result of a group. ATB, active tuberculosis; LTBI, latent tuberculosis infection; HC, healthy controls.

We characterized lymphocyte phenotype in ATB, LTBI, and HC. Most of the phenotypes did not significantly differ between ATB and non-ATB. Statistical differences were only found in HLA-DR expression on T cells and the proportion of Treg. Specifically, the proportions of HLA-DR^+^CD3^+^ T cells and Treg in ATB patients were significantly higher than those in LTBI individuals or HC (**Figure 3**). The proportions of CD28^+^CD4^+^ T cells, CD28^+^CD8^+^ T cells, HLA-DR^+^CD3^+^CD4^+^ T cells, HLA-DR^+^CD3^+^CD8^+^ T cells, CD45RA^+^CD4^+^ T cells, CD45RO^+^CD4^+^ T cells, and CD45RO^+^ Treg of participants with ATB did not differ significantly from LTBI or HC (**Figure 3**). No statistical difference was observed in all indexes among lymphocyte phenotype analysis between LTBI and HC (**Figure 3**).

### Lymphocyte Function in ATB, LTBI, and HC

**Figure 4:**
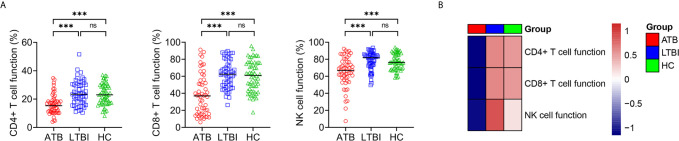
The results of lymphocyte function in ATB, LTBI, and HC. **(A)** Scatter plots showing the results of lymphocyte function in ATB (n = 54), LTBI (n = 57), and HC (n = 60). Horizontal lines indicate the median. ***P < 0.001, ns, no significance (Mann-Whitney *U* test). **(B)** Heatmap showing the results of lymphocyte function in ATB group, LTBI group, and HC group. Each rectangle indicates the median result of a group. ATB, active tuberculosis; LTBI, latent tuberculosis infection; HC, healthy controls.

Lymphocyte function was investigated in ATB, LTBI, and HC. It was found that the function of CD4^+^ T cells, CD8^+^ T cells, and NK cells was significantly lower in ATB patients than in LTBI individuals or HC, while no significant difference presented in CD4^+^ T cell function, CD8^+^ T cell function, and NK cell function between LTBI and HC group (**Figure 4**).

### Establishing Immunodiagnostic Model for Stratifying the Status of MTB Infection

**Figure 5:**
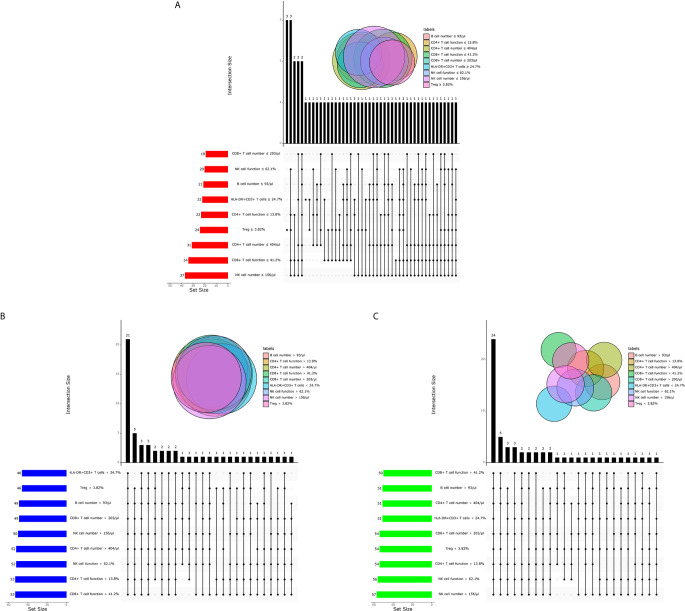
The cross set between various immune indicators in ATB, LTBI, and HC. **(A)** Upset plot showing the cross set between various immune indicators in ATB. **(B)** Upset plot showing the cross set between various immune indicators in LTBI. **(C)** Upset plot showing the cross set between various immune indicators in HC. ATB, active tuberculosis; LTBI, latent tuberculosis infection; HC, healthy controls.

In order to investigate the possibility of combining different immune indicators to distinguish the status of MTB infection, we performed heatmap analysis and discovered the potential of combination of these indexes to distinguish ATB from non-ATB (**Supplementary Figure 2**). We next analyzed the cross set of indicators with significant differences in three groups. The overlap of 9 indicators with significant difference indicated the possible conjunct use for stratification (**Figure 5**).

**Figure 6:**
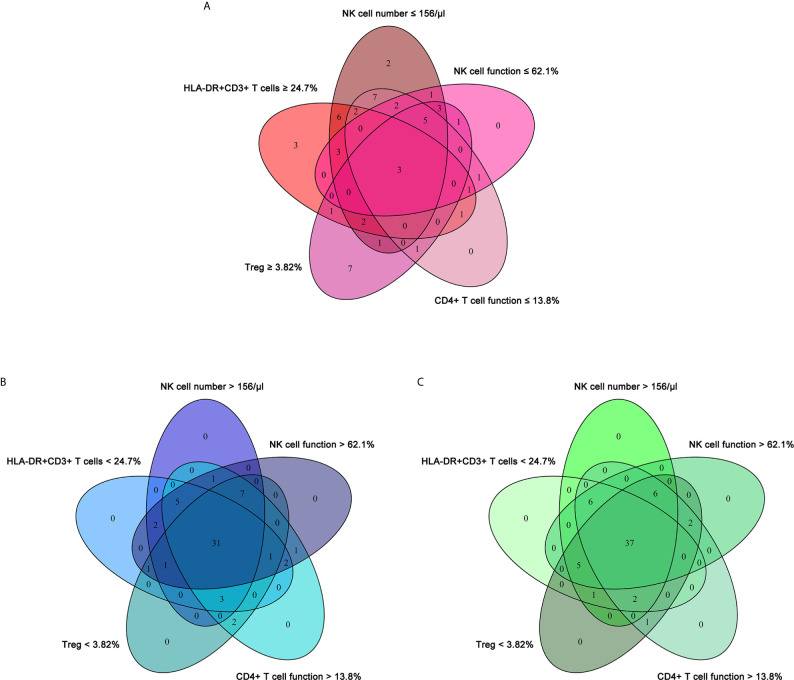
The cross set between various immune indicators in ATB, LTBI, and HC. **(A)** Venn diagrams showing the overlap of NK cell number, HLA-DR^+^CD3^+^ T cells, Treg, CD4^+^ T cell function, and NK cell function in ATB patients. **(B)** Venn diagrams showing the overlap of NK cell number, HLA-DR^+^CD3^+^ T cells, Treg, CD4^+^ T cell function, and NK cell function in LTBI individuals. **(C)** Venn diagrams showing the overlap of NK cell number, HLA-DR^+^CD3^+^ T cells, Treg, CD4^+^ T cell function, and NK cell function in HC. ATB, active tuberculosis; LTBI, latent tuberculosis infection; HC, healthy controls.

**Figure 7:**
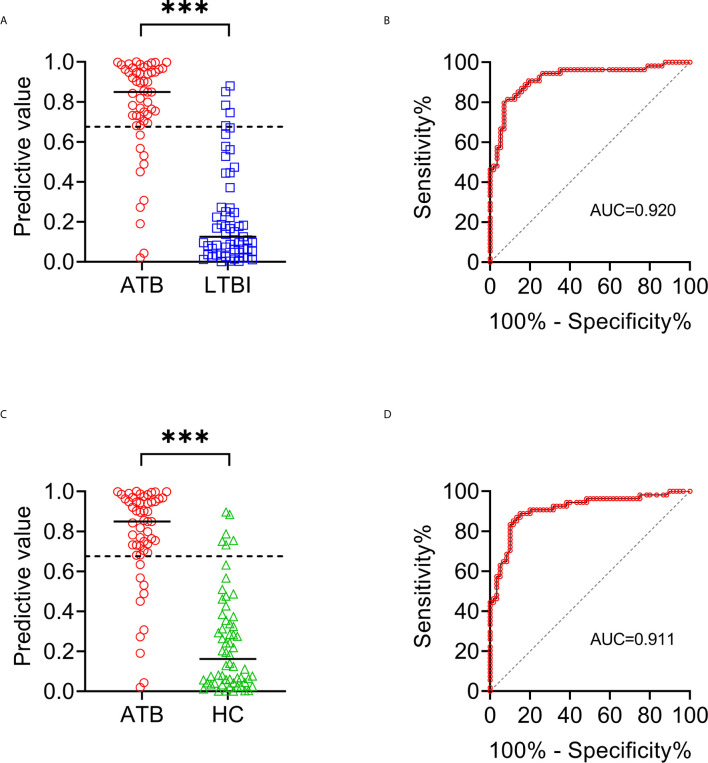
The performance of established diagnostic model for distinguishing ATB from LTBI and HC. **(A)** Scatter plots showing the predictive value of diagnostic model in ATB patients (n = 54) and LTBI individuals (n = 57). Horizontal lines indicate the median. ***P < 0.001 (Mann-Whitney *U* test). Dotted line indicates the cutoff value in distinguishing these two groups. **(B)** ROC analysis showing the performance of diagnostic model in discriminating ATB patients from LTBI individuals. **(C)** Scatter plots showing the predictive value of diagnostic model in ATB patients (n = 54) and HC (n = 60). Horizontal lines indicate the median. ***P < 0.001 (Mann-Whitney *U* test). Dotted line indicates the cutoff value in distinguishing these two groups. **(D)** ROC analysis showing the performance of diagnostic model in discriminating ATB patients from HC. ATB, active tuberculosis; LTBI, latent tuberculosis infection; HC, healthy controls; ROC, receiver operating characteristic.

**Figure 8:**
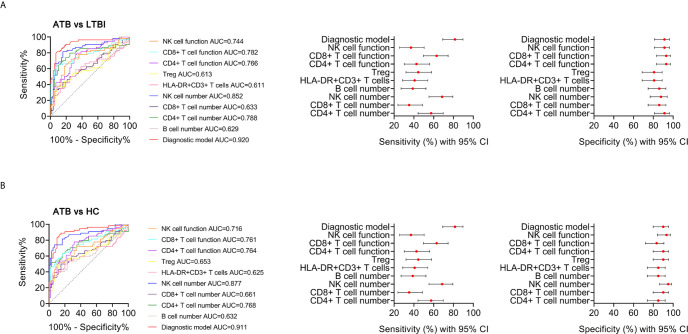
The performance of various indicators in discriminating ATB from LTBI and HC. **(A)** ROC analysis showing the performance of various indicators in discriminating ATB patients from LTBI individuals. Liner plots showing the sensitivity and specificity of different indicators as well as their 95% CI. **(B)** ROC analysis showing the performance of various indicators in discriminating ATB patients from HC. Liner plots showing the sensitivity and specificity of different indicators as well as their 95% CI. ATB, active tuberculosis; LTBI, latent tuberculosis infection; HC, healthy controls; AUC, area under the curve; CI, confidence interval.

To establish the diagnostic model based on a combination for differentiating ATB from LTBI, all variables with statistical significance were used for multivariable logistic regression analysis. The diagnostic model was established as the follows: P = 1/[1 + e^-(-0.005* NK cell number + 0.102 * HLA-DR+CD3+ T cells + 0.53^*^Treg - 0.147 * CD4+ T cell function - 0.049 * NK cell function + 3.95)^] P, predictive value; e, natural logarithm. Venn diagram showed the overlap of these five parameters in ATB, LTBI, and HC groups and confirmed the appropriate combination of them (**Figure 6**). The AUC presented by the diagnostic model was 0.920 (95% CI, 0.867-0.973) (**Table 2** and **Figures 7A, B** **)**. The cutoff value of 0.676 for diagnostic model showed a sensitivity of 81.48% (95% CI, 69.16%-89.62%) and specificity of 91.23% (95% CI, 81.06%-96.20%) in distinguishing between ATB and LTBI (**Table 2**). We also applied the model to discriminate ATB from HC. It was observed that the sensitivity and specificity for the model were 81.48% (95% CI, 69.16%-89.62%) and 90.00% (95% CI, 79.85%-95.34%) with the threshold as 0.676 (**Table 3** and **Figures 7C, D** **)**. Meanwhile, the comparison between AUCs showed that the performance of the diagnostic model was superior to the individual immune indicator (**Tables 2**, **3** and **Figure 8**).

**Table 2:** The performance of different methods for distinguishing between ATB and LTBI.

Methods	Cutoff value	AUC (95% CI)	Sensitivity (95% CI)	Specificity (95% CI)	PPV (95% CI)	NPV (95% CI)	PLR (95% CI)	NLR (95% CI)	Accuracy
CD4^+^ T cell number (/μl)	404	0.788^†^ (0.694-0.882)	57.41% (44.16%-69.67%)	91.23% (81.06%-96.20%)	86.11% (71.34%-93.92%)	69.33% (58.17%-78.61%)	6.54 (2.75-15.59)	0.47 (0.34-0.64)	74.77%
CD8^+^ T cell number (/μl)	203	0.633^‡^ (0.528-0.737)	35.19% (23.82%-48.52%)	85.96% (74.68%-92.71%)	70.37% (51.52%-84.15%)	58.33% (47.65%-68.29%)	2.51 (1.2-5.24)	0.75 (0.6-0.94)	61.26%
NK cell number (/μl)	156	0.852^*^ (0.778-0.927)	68.52% (55.26%-79.32%)	87.72% (76.75%-93.92%)	84.09% (70.63%-92.07%)	74.63% (63.07%-83.51%)	5.58 (2.72-11.43)	0.36 (0.24-0.54)	78.38%
B cell number (/μl)	93	0.629^‡^ (0.523-0.736)	38.89% (27.04%-52.21%)	85.96% (74.68%-92.71%)	72.41% (54.28%-85.30%)	59.76% (48.94%-69.70%)	2.77 (1.34-5.72)	0.71 (0.56-0.9)	63.06%
HLA-DR^+^CD3^+^ T cells (%)	24.7	0.611^‡^ (0.504-0.719)	40.74% (28.68%-54.03%)	80.70% (68.66%-88.87%)	66.67% (49.61%-80.25%)	58.97% (47.89%-69.22%)	2.11 (1.13-3.93)	0.73 (0.57-0.95)	61.26%
Treg (%)	3.82	0.613^‡^ (0.506-0.720)	44.44% (32.00%-57.62%)	80.70% (68.66%-88.87%)	68.57% (52.02%-81.45%)	60.53% (49.29%-70.75%)	2.3 (1.25-4.23)	0.69 (0.53-0.9)	63.06%
CD4^+^ T cell function (%)	13.8	0.766^‡^ (0.678-0.854)	42.59% (30.33%-55.84%)	92.98% (83.30%-97.24%)	85.19% (67.52%-94.09%)	63.10% (52.42%-72.63%)	6.07 (2.25-16.41)	0.62 (0.49-0.79)	68.47%
CD8^+^ T cell function (%)	41.2	0.782^†^ (0.692-0.873)	62.96% (49.63%-74.58%)	92.98% (83.30%-97.24%)	89.47% (75.87%-95.83%)	72.60% (61.44%-81.51%)	8.97 (3.41-23.59)	0.4 (0.28-0.57)	78.38%
NK cell function (%)	62.1	0.744^‡^ (0.650-0.838)	37.04% (25.42%-50.37%)	91.23% (81.06%-96.20%)	80.00% (60.87%-91.14%)	60.47% (49.90%-70.14%)	4.22 (1.71-10.45)	0.69 (0.55-0.86)	64.86%
Diagnostic model	0.676	0.920 (0.867-0.973)	81.48% (69.16%-89.62%)	91.23% (81.06%-96.20%)	89.80% (78.24%-95.56%)	83.87% (72.79%-91.00%)	9.29 (3.98-21.66)	0.2 (0.12-0.36)	86.49%

^*^Compared with diagnostic model using z statistic, P < 0.05; ^†^compared with diagnostic model using z statistic, P < 0.01; ^‡^compared with diagnostic model using z statistic, P < 0.001; ATB, active tuberculosis; LTBI, latent tuberculosis infection; AUC, area under the curve; PPV, positive predictive value; NPV, negative predictive value; PLR, positive likelihood ratio; NLR, negative likelihood ratio; CI, confidence interval.

**Table 3:** The performance of different methods for distinguishing between ATB and HC.

Methods	Cutoff value	AUC (95% CI)	Sensitivity (95% CI)	Specificity (95% CI)	PPV (95% CI)	NPV (95% CI)	PLR (95% CI)	NLR (95% CI)	Accuracy
CD4^+^ T cell number (/μl)	404	0.768^†^ (0.675-0.862)	57.41% (44.16%-69.67%)	85.00% (73.89%-91.90%)	77.50% (62.50%-87.69%)	68.92% (57.66%-78.31%)	3.83 (2.01-7.29)	0.5 (0.36-0.7)	71.93%
CD8^+^ T cell number (/μl)	203	0.661^‡^ (0.558-0.764)	35.19% (23.82%-48.52%)	90.00% (79.85%-95.34%)	76.00% (56.57%-88.51%)	60.67% (50.29%-70.18%)	3.52 (1.52-8.16)	0.72 (0.58-0.89)	64.04%
NK cell number (/μl)	156	0.877 (0.809-0.945)	68.52% (55.26%-79.32%)	95.00% (86.30%-98.29%)	92.50% (80.14%-97.42%)	77.03% (66.25%-85.13%)	13.7 (4.48-41.9)	0.33 (0.22-0.49)	82.46%
B cell number (/μl)	93	0.632^‡^ (0.525-0.738)	38.89% (27.04%-52.21%)	85.00% (73.89%-91.90%)	70.00% (52.12%-83.34%)	60.71% (50.02%-70.47%)	2.59 (1.3-5.16)	0.72 (0.57-0.91)	63.16%
HLA-DR^+^CD3^+^ T cells (%)	24.7	0.625^‡^ (0.517-0.733)	40.74% (28.68%-54.03%)	85.00% (73.89%-91.90%)	70.97% (53.41%-83.91%)	61.45% (50.69%-71.19%)	2.72 (1.37-5.38)	0.7 (0.55-0.89)	64.04%
Treg (%)	3.82	0.653^‡^ (0.548-0.758)	44.44% (32.00%-57.62%)	90.00% (79.85%-95.34%)	80.00% (62.70%-90.50%)	64.29% (53.62%-73.70%)	4.44 (1.97-10.05)	0.62 (0.48-0.8)	68.42%
CD4^+^ T cell function (%)	13.8	0.764^‡^ (0.676-0.852)	42.59% (30.33%-55.84%)	90.00% (79.85%-95.34%)	79.31% (61.61%-90.16%)	63.53% (52.92%-72.97%)	4.26 (1.88-9.67)	0.64 (0.5-0.81)	67.54%
CD8^+^ T cell function (%)	41.2	0.761^‡^ (0.669-0.852)	62.96% (49.63%-74.58%)	83.33% (71.97%-90.69%)	77.27% (63.01%-87.16%)	71.43% (59.95%-80.68%)	3.78 (2.07-6.89)	0.44 (0.31-0.64)	73.68%
NK cell function (%)	62.1	0.716^‡^ (0.620-0.813)	37.04% (25.42%-50.37%)	93.33% (84.08%-97.38%)	83.33% (64.15%-93.32%)	62.22% (51.90%-71.54%)	5.56 (2.03-15.23)	0.67 (0.54-0.84)	66.67%
Diagnostic model	0.676	0.911 (0.855-0.967)	81.48% (69.16%-89.62%)	90.00% (79.85%-95.34%)	88.00% (76.20%-94.38%)	84.38% (73.57%-91.29%)	8.15 (3.77-17.59)	0.21 (0.12-0.36)	85.96%

^†^Compared with diagnostic model using z statistic, P < 0.01; ^‡^compared with diagnostic model using z statistic, P < 0.001; ATB, active tuberculosis; HC, healthy controls; AUC, area under the curve; PPV, positive predictive value; NPV, negative predictive value; PLR, positive likelihood ratio; NLR, negative likelihood ratio; CI, confidence interval.

### The Relationship Between Immune Indicators in ATB Patients

**Figure 9:**
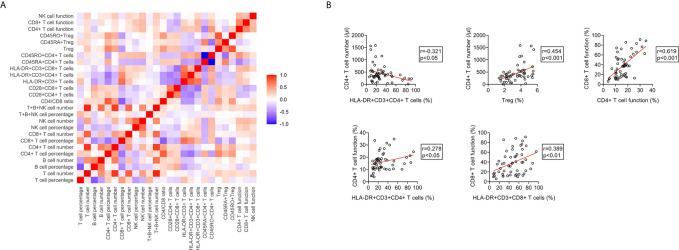
The relationship between different immune indicators in ATB patients. **(A)** Heatmap showing the relationship different immune indicators in ATB patients. **(B)** Scatter plots showing the correlation between the number, phenotype, and function of lymphocytes in ATB patients. Each symbol represents an individual donor. ATB, active tuberculosis.

We conducted correlation analysis of different immune indicators in ATB patients (**Figure 9A**). It was observed that the proportion of HLA-DR^+^CD3^+^CD4^+^ T cells was significantly negative, whereas the proportion of Treg was significantly positive, with the number of CD4^+^ T cells. There was a significantly positive correlation between the function of CD4^+^ T cells and the expression of HLA-DR on these cells. The same phenomenon was also presented in CD8^+^ T cells. Meanwhile, statistically positive correlation existed between CD4^+^ T cell function and CD8^+^ T cell function (**Figure 9B**).

## Discussion

Control of the TB pandemic remains hindered (36–38). Major challenges for TB control include the lack of specific drugs and biomarkers for stratifying MTB infection, and the emergence of drug resistance (39–46). Current gold standard diagnostics that rely on bacteriological assays are slow and challenging to implement, as well as incompatible with the healthcare settings in which TB is frequently seen (47, 48). On the other hand, although many efforts including various omics have been made to overcome the issue, these methods have not been effectively verified, making it difficult to transform into clinical practice. Hence, the stratification of MTB infection still needs to be addressed with urgency.

Immune biomarkers based on flow cytometer have recently begun to emerge as clinically useful diagnostic and prognostic markers of infectious disease (49–51). Growing evidence has demonstrated that TB may elicit specific patterns of immune response (52–54). Nonetheless, there was rare study targeted for comprehensive evaluation for host immunity towards MTB infection. Most previous studies focused on the number of lymphocyte or its subsets in ATB. A few studies explored the immunophenotype of ATB patients, while few studies evaluated lymphocyte function of subjects with MTB infection. Thus, these previous studies have not fully clarified the host immune landscape among subjects with MTB infection on account of methodological limitations. Our study simultaneously determined the immune characteristics of lymphocyte at different stages of MTB infection from number, phenotype, and function for the first time. We confirmed the low levels of lymphocyte number and function, hyperactivation and high proportion of Treg in patients with ATB. These data indicate that ATB patients are in a state of hyperinflammatory but with low immune potential. TB is generally considered to be a disease with malnutrition. Some previous studies have reported the low level of serum iron (55) and prealbumin (56) in ATB patients. Thus, the low level of lymphocyte number and function found in our research echoed these phenomena. Furthermore, we discovered the potential of the combination of three types of immune indicators to differentiate the status of MTB infection through Venn diagram analysis, and successfully established an immunodiagnostic model using logistic regression. The model based on the combination of NK cell number, HLA-DR^+^CD3^+^ T cells, Treg, CD4^+^ T cell function, and NK cell function could efficaciously distinguish ATB from LTBI and HC.

Some publications have shown that the phenotype including HLA-DR, CD38, and Ki-67 on TB-specific cells was helpful for TB diagnosis (28, 57). However, this type of method requires additional specific stimulation for more than 12 hours. Besides, in order to obtain enough IFN-γ^+^ or TNF-α^+^ cells for subgroup analysis, a large volume of peripheral blood is usually needed (57). The complexity of these operations makes it difficult into clinical transformation. In addition, owing to the existence of ATB patients with negative T-SPOT results and MTB infected individuals with low-value-T-SPOT results (58–61), the effectiveness of this method will be greatly reduced due to not getting enough TB-specific cells for analysis. On the other hand, some literature reported that cytokines including IL-2, IFN-γ, and TNF-α have the potential to diagnose TB (62–65). However, the value of most unstimulated cytokines was limited, the more advantageous diagnostic utility often also requires TB-specific stimulation. Moreover, the large heterogeneity between different studies also hinders the possibility of its translation into clinical practice (66). The detection of lymphocyte-related indicators that we performed in the present study requires only a small volume of peripheral blood plus short-term non-specific stimulation, while eliminating cumbersome extraction of peripheral blood mononuclear cells. Therefore, our established diagnostic model has more advantages in applying to clinical practice.

Regarding the indicators observed in this study, the immune profiles did not differ significantly between LTBI and HC groups. On the one hand, these data indicates that the host immunity of individuals with LTBI may temporarily successfully resist MTB. As a result, the body shows no immune barriers or defects as a whole. On the other hand, it may be that the immune indicators observed in our research are not specific and comprehensive, they cannot reflect the subtle difference of immune characteristics between the two groups. Various immune cell population including monocytes, dendritic cells, neutrophils need to be further analyzed in a broader spectrum. Meanwhile, detailed classification such as helper T cell and follicular helper T cell should be also conducted. These directions are also applicable to the expansion of immune observation in ATB group.

Several limitations should be noticed in the current study. First, the sample size in this study is relatively small, and stratified analysis targeted for different underlying diseases such as HIV infection has not been carried out. Validation by larger population in areas with different disease burdens would be further needed. Second, lymphocyte immune indicators analyzed in this study are not comprehensive enough, and multi-dimensional analysis using polychromatic flow cytometry is also very necessary. Third, given that time course comparisons under treatment, MTB-specific assays, and identified immune cell markers such as CD38 and CD27 were missing in the present study (67, 68), further investigation targeting monitoring or conjunction of different methods are needed. Fourth, since the underlying diseases might affect the levels of these lymphocyte-related immune indicators, individuals with other infectious diseases, tumors, and autoimmune diseases were excluded from this study. More exploration targeting the effect of these underlying diseases on our established model should be conducted in the future. Eventually, the present study only focuses on the characteristics of lymphocytes among MTB infection. Other immune cells including B cells and dendritic cells are also proved involved in the pathogenesis of TB (69–72). Therefore, different types of immune cells should be also included for a more comprehensive analysis.

In conclusion, our findings suggests that the diagnostic model based on the combination of lymphocyte-related indicators may be an adjunctive but useful method in the diagnosis of TB.

## Data Availability Statement

The original contributions presented in the study are included in the article/**Supplementary Material**. Further inquiries can be directed to the corresponding authors.

## Ethics Statement

The studies involving human participants were reviewed and approved by the ethics committee of Tongji Hospital, Tongji Medical College, Huazhong University of Science and Technology. The patients/participants provided their written informed consent to participate in this study.

## Author Contributions

YL and YX designed and oversaw the study; QL and GT contributed to lymphocyte function assay; HS and WL contributed to lymphocyte subset analysis; LM conducted lymphocyte phenotype analysis; XY, YuZ, ZC, YaZ, WYL, SW, FW, and ZS coordinated data collection and management. YL and YC did the statistical analysis. YL wrote the manuscript. All authors contributed to the article and approved the submitted version.

## Funding

This work was funded by Graduate Innovation Fund of Huazhong University of Science and Technology (grant number 2021yjsCXCY088), National Mega Project on Major Infectious Disease Prevention of China (grant number 2017ZX10103005-007), and the National Natural Science Foundation (grant number 81401639 and 81902132).

## Conflict of Interest

The authors declare that the research was conducted in the absence of any commercial or financial relationships that could be construed as a potential conflict of interest.

## Supplementary Material

Supplementary Figure 1 Representative pictures showing the negative and positive results of T-SPOT assay. The number in the upper left corner of each graph indicates the number of spot-forming cells in each well. ESAT-6, early secreted antigenic target 6; CFP-10, culture filtrate protein 10; PHA, phytohemagglutinin.

Supplementary Figure 2 The cluster analysis of immune indicators in ATB, LTBI, and HC. **(A)** Heatmap showing the cluster analysis of lymphocyte subsets, phenotype, and function in ATB patients (n = 54) and LTBI individuals (n = 57). Each rectangle indicates a result of a subject. **(B)** Heatmap showing the cluster analysis of lymphocyte subsets, phenotype, and function in ATB patients (n = 54) and HC (n = 60). Each rectangle indicates a result of a subject. ATB, active tuberculosis; LTBI, latent tuberculosis infection; HC, healthy controls.
