# Supplementary figures and images for: Lymphocyte-Related Immunological Indicators for Stratifying Mycobacterium tuberculosis Infection
Source: Front Immunol. 2021 Jun 30;12:658843. doi: 10.3389/fimmu.2021.658843 (PMC8278865; doi:10.3389/fimmu.2021.658843)

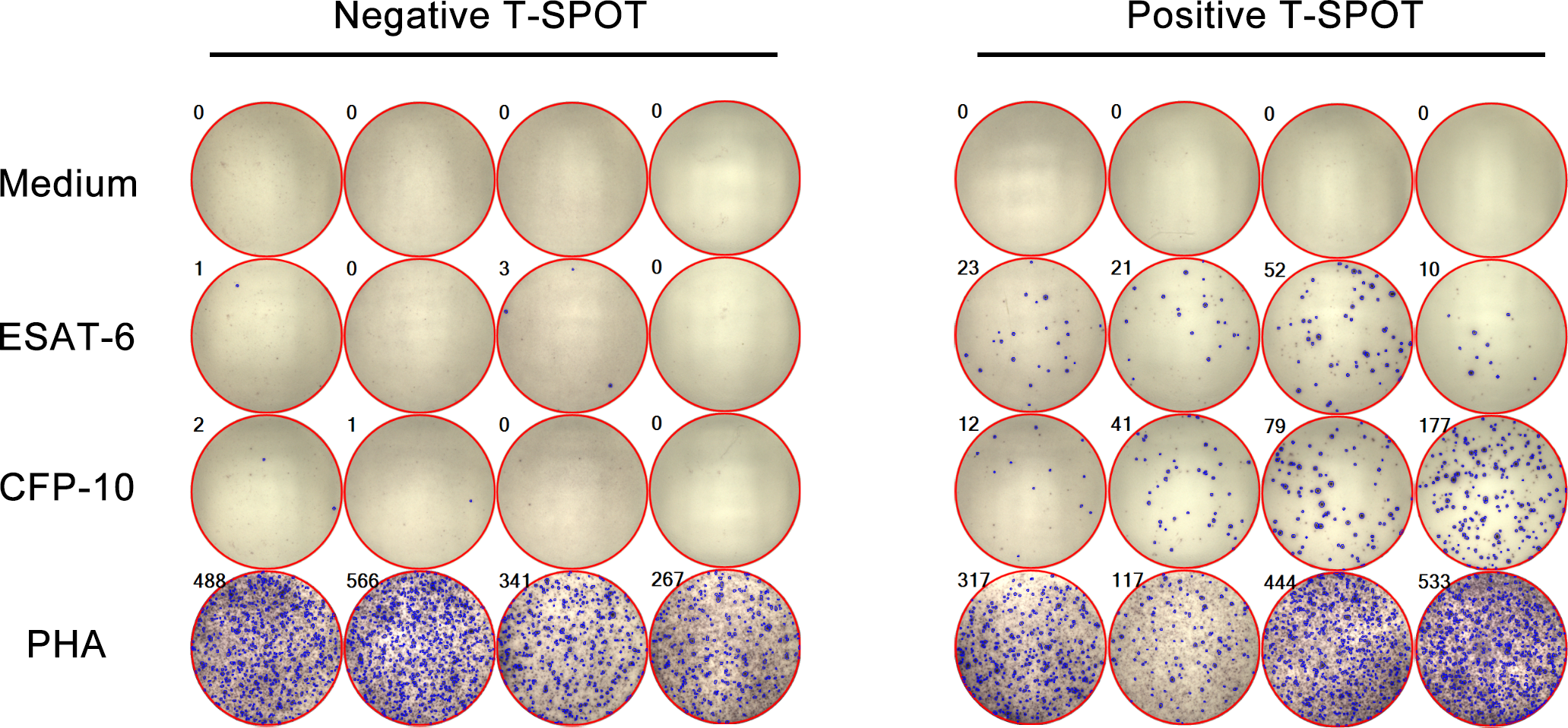

Supplement: Supplementary Figure 1 — Representative pictures showing the negative and positive results of T-SPOT assay. The number in the upper left corner of each graph indicates the number of spot-forming cells in each well. ESAT-6, early secreted antigenic target 6; CFP-10, culture filtrate protein 10; PHA, phytohemagglutinin. [file Image_1.tif]
